# Vitamin E – a scoping review for Nordic Nutrition Recommendations 2023

**DOI:** 10.29219/fnr.v67.10238

**Published:** 2023-10-12

**Authors:** Essi Hantikainen, Ylva Trolle Lagerros

**Affiliations:** 1 Institute for Biomedicine, Eurac Research, Bolzano, Italy; 2 Division for Clinical Epidemiology, Department of Medicine (Solna), Karolinska Institutet, Stockholm, Sweden

**Keywords:** vitamin E, tocopherol, antioxidants, recommendations

## Abstract

Current evidence suggests that dietary vitamin E (as α-tocopherol) intake has a beneficial role in preventing certain chronic diseases. In contrast, there is no clear evidence for the benefit of α-tocopherol supplements in a generally healthy population. Deficiency symptoms are rare and mainly occur due to genetic or other factors affecting α-tocopherol absorption and/or metabolism, rather than a low α-tocopherol intake. No qualified systematic review was identified by the NNR2023 project for updating the dietary reference values (DRV).

## Popular scientific summary

- Vitamin E is a fat-soluble antioxidant that protects cell membranes and lipoproteins in plasma from oxidative damage.
- Other functions include modulation of gene expression, inhibition of cell proliferation and regulation of bone mass.
- There are eight natural forms of vitamin E; only α-tocopherol meets human requirements.
- Foods rich in vitamin E include vegetable oils, fat spreads, nuts, seeds and egg yolk, but cereal products also contribute in Nordic and Baltic diets.
- Vitamin E deficiency can be caused by fat malabsorption.
- Animal and observational studies suggest possible beneficial effects on some chronic diseases, but the evidence is limited, and clinical trials are lacking.

Vitamin E is a liposoluble antioxidant that not only prevents the propagation of free radicals in membranes and in plasma lipoproteins (1) but also exhibits non-antioxidant activities, such as modulation of gene expression, inhibition of cell proliferation and regulation of bone mass (2). Although plants make eight different forms of vitamin E, namely four tocopherols (α-, β-, γ- and δ) and four tocotrienols (α-, β-, γ- and γ), only α-tocopherol meets human vitamin E requirements according to the US National Academy of Sciences, Engineering, and Medicine (NASEM; formerly the Institute of Medicine) (3). α-Tocopherol is the only form found to reverse vitamin E deficiency symptoms in humans, including neuropathy, haemolytic anaemia and the progressive disease ataxia with vitamin E deficiency (AVED), which is due to genetic defects in the α-tocopherol transfer protein (α-TTP) (3). Several studies suggest that besides α-tocopherol, other tocopherols and tocotrienols might have important functions and beneficial effects on various chronic disease outcomes (4, 5). However, as evidence of their importance in human health still is limited, the dietary reference values are therefore confined to α-tocopherol.

The naturally occurring α-tocopherol in foods is the stereoisomer RRR-α-tocopherol. Synthetic forms of α-tocopherol are present in fortified foods. α-Tocopherol supplements are sold as esters of either the natural RRR or the synthetic mixture (also known as all-rac-α-tocopherol) that contains an equal mixture of eight different stereoisomers, of which four stereoisomers are in the 2R-stereoisomeric form (RRR-, RSR-, RRS- and RSS-α-tocopherol) and four are in the 2S-stereoisomeric form (SRR- SSR-, SRS- and SSS-α-tocopherol) (6). All of the stereoisomers have equal antioxidative activities, but only those with the 2R-configuration (RRR, RSR, RRS and RSS) have biologically relevant activities. Due to the lower affinity that α-tocopherol transport protein (α-TTP) has for 2S-isomers, the relative bioavailability of the synthetic form of α-tocopherol is suggested to be only half that of the naturally occurring α-tocopherol (7). This means that only α-tocopherol in foods and 2R-α-tocopherols in vitamin E preparations contribute to vitamin E activity, which traditionally is expressed in terms of α-tocopherol equivalents (α-TE) and includes the small amounts of activity suggested by animal experiments to be provided by other tocopherol. For commercially available α-tocopherol preparations, the following conversion factors to α-TE have been suggested: 0.5 for all-rac-α-tocopherol, 0.455 for all-rac-α-tocopheryl acetate and 0.91 for RRR-α-tocopheryl acetate (8, 9). In older literature, vitamin E activity was expressed as IUs. One international unit (IU) is equivalent to 0.67 mg of the natural form and 0.45 mg of the synthetic form of the vitamin (10).

Findings from animal or observational studies suggest α-tocopherol to be anti-atherosclerotic, anti-cancerogenic, anti-allergic, anti-lipidemic, anti-diabetic, antihypertensive, anti-inflammatory and anti-obesogenic. On the other hand, these results have still to be confirmed by clinical trials in people with adequate nutritional status (11).

A global review reported that 82% of mean and median data points of α-tocopherol were below the recommended daily allowance (RDA) of 15 mg/day set by NASEM. The corresponding figure was 61% for the estimated average requirement (EAR) of 12 mg/day, across all populations aged 14 years and older (12).

The following scoping review will focus on the current α-tocopherol status and requirements in the Nordic and Baltic countries.

## Methods

This scoping review follows the protocol developed within the NNR2023 project (13). Initially, no qualified systematic review was identified by the NNR2023 project (14). The literature search was performed in PubMed and updated June 28, 2022. The authors individually reviewed the results from the initial database search. Firstly, authors screened the titles to select abstracts; thereafter, abstracts were reviewed to choose potential full-length articles to review. Disagreements between authors were solved before moving on to the next review step.

**Fig. 1:**
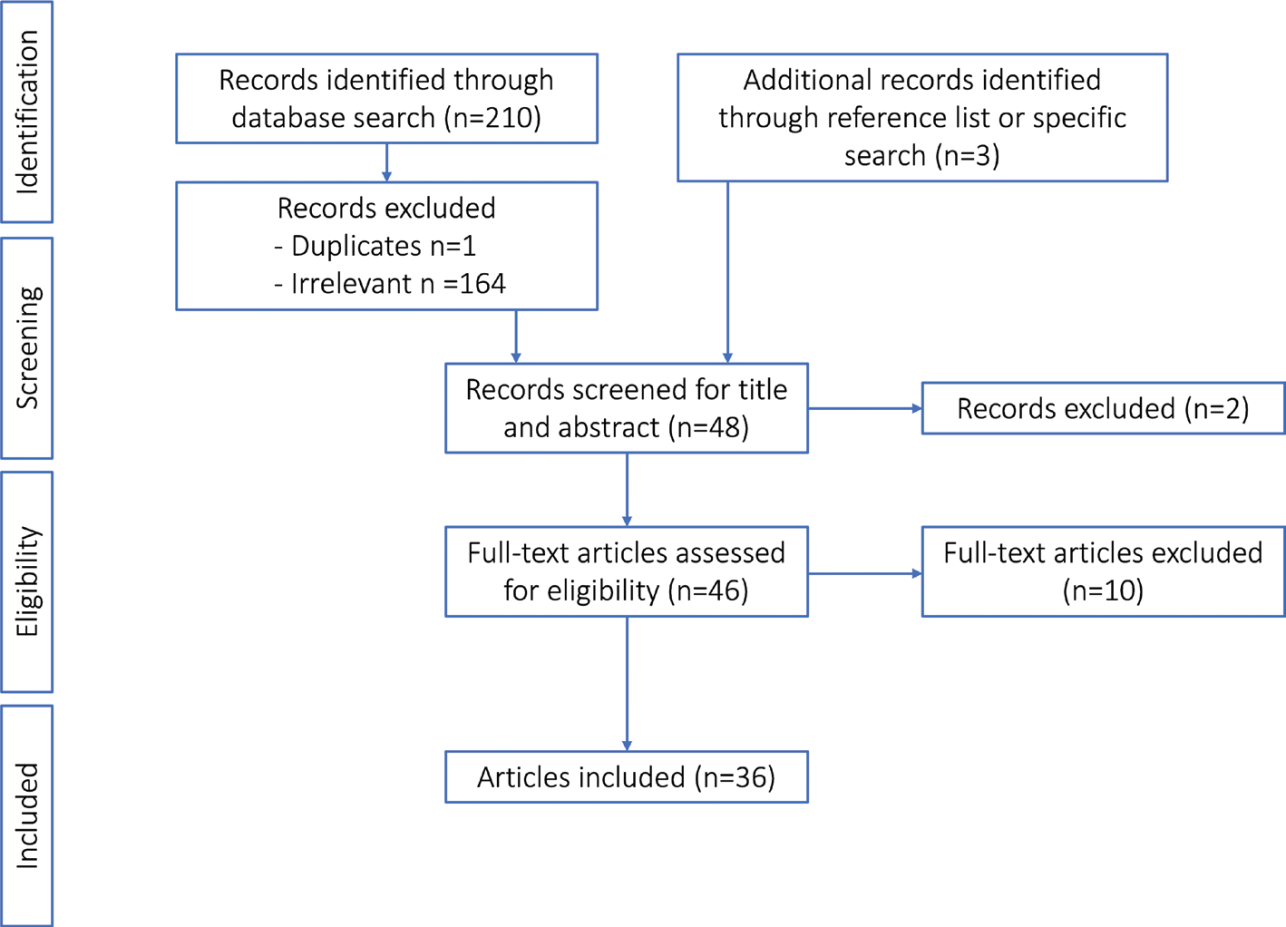
Flow chart for the selection procedure of relevant literature for vitamin E and health-related outcomes in the NNR2022.

The search string below was used to include systematic reviews and meta-analyses to select relevant literature for vitamin E and health-related outcomes. Articles were included if dietary or supplemental vitamin E with focus on α-tocopherol was the exposure of interest and if the outcome was relevant for Nordic and Baltic countries. Studies were also considered if the article focused on relevant subgroups of the population (e.g. pregnant/lactating women and children). A flowchart of the literature search is presented in Fig. 1.

### Search string

(“vitamin e”[MeSH Terms] OR “vitamin e”[ Title]) AND (“2011”[PDAT]: “3000”[PDAT]) AND Humans[Filter] AND (meta-analysis[Filter] OR systematicreview[Filter]) AND “humans”[MeSH Terms])

All articles were checked for quality using the NNR-adjusted AMSTAR2 checklist (13). Although not all articles were compliant with the checklist, they could be included if no other reference was available. For most of the health outcomes, there were only a limited number of systematic reviews and meta-analyses available, which were based on randomised controlled trials. Therefore, for some of the health outcomes, we additionally searched for meta-analyses and systematic reviews or single Randomized Controlled Trial (RCT)s using snowballing technique and citation search. If no articles were found based on these techniques, a specific search related to the outcome was conducted. A detailed description of the quality check is provided in Supplementary Table 1.

## Physiology and metabolism

The uptake, transport and tissue delivery of α-tocopherol involves molecular, biochemical and cellular processes that are closely related to overall lipid and lipoprotein metabolism (15). The absorption efficiency of α-tocopherol shows large variability with values ranging between 10 and 79% (2). In the gastrointestinal tract, α-tocopherol requires biliary and pancreatic secretions in order to form micelles for subsequent uptake by the intestine (6). As α-tocopherol transfers to mixed micelles, the transport across the intestinal cell is influenced by several factors, which might explain the observed variations regarding α-tocopherol absorption efficiency. The presence of fat is required for an efficient absorption and the amount of fat provided in the meal determines α-tocopherols bio-accessibility, as fat facilitates its extraction from the food matrix, stimulates biliary secretion and promotes micelle formation (16). It is further noteworthy mentioning that genetic factors including polymorphisms in genes coding for α-tocopherol and lipid intestinal metabolism have been associated with a modulation of α-tocopherol bioavailability in humans (16).

Initially, it was thought that α-tocopherol absorption occurred only by passive diffusion through the enterocyte membrane, but it turned out that several groups of transmembrane proteins (i.e. NPC1L1, SR-BI which also are involved in the cholesterol transport), play a key role in the intestinal absorption of α-tocopherol (11). The two absorption mechanisms, that is, protein-mediated absorption and passive absorption, might be complementary, with protein-mediated absorption occurring mainly at dietary doses and passive diffusion at pharmacological doses (2). After intestinal absorption, α-tocopherol is incorporated into chylomicrons via the apolipoprotein B pathway (11). Chylomicrons transport α-tocopherol from the intestine through the circulation to the liver (17), which is the main regulator of the body’s α-tocopherol levels as it not only controls α-tocopherol concentrations but also appears to be the major site of α-tocopherol metabolism and excretion (6, 16). In the liver, out of all vitamin E forms, the protein α-TTP preferentially binds to α-tocopherol and is responsible for its further transportation, while the remaining tocochromanols are metabolised and excreted in the bile. Specifically, α-TTP mediates the incorporation of α-tocopherol into very low-density lipoproteins (VLDL) and supports the secretion of these complexes back into the circulation. VLDLs in the blood are further catabolised to low (LDL) and high-density (HDL) lipoproteins. α-Tocopherol that is delivered into LDL lipoproteins is transferred to the tissues, where it performs its functions (11). Unlike vitamins A and D, α-tocopherol does not accumulate to toxic levels in the liver or extrahepatic tissues (17). About 90% of the total amount of all vitamin E forms is accumulated in the adipose tissue, mainly in adipocyte lipid droplets, and consists of two-thirds of α-tocopherol and one-third of y-tocopherol (11).

α-Tocopherol is metabolised similarly to xenobiotics. It is initially ω-oxidised and thereafter undergoes several rounds of β-oxidation. Metabolites are conjugated and excreted with bile and urine. As a result of these various mechanisms, liver α-tocopherol concentrations are closely regulated, and therefore potential adverse α-tocopherol effects are limited (6). The rates of α-tocopherol entering or leaving the plasma are dependent on absorption, tissue delivery and excretion. The apparent half-life of RRR-α-tocopherol in plasma of normal subjects was found to be between 48 h up to 60 h (6).

The main biochemical function of α-tocopherol has been suggested to be its antioxidant activity. α-Tocopherol is present in cell membranes. It has a significant preventive role in the oxidative damage of molecules such as DNA or lipids by neutralising free radicals and breaking the chain reaction in the oxidation of the polyunsaturated fatty acids (11). In addition, several other important biological functions, including modulation of cell signaling and gene expression, have been described (18). α-Tocopherol further modulates the activity of several enzymes. By comparing α-tocopherol with other antioxidants, it was concluded that cell proliferation inhibition by α-tocopherol was not caused by its antioxidant properties. Instead, it seemed to be caused by a non-antioxidant mechanism, such as inhibition of the activity of protein kinase C (PKC), which is fundamental for α-tocopherol protection against inflammation, lipid deposition in aorta, diabetic vascular complications and platelet aggregation (19). Several possible regulatory pathways have been described for α-tocopherol, which can alter the activity of transcription factors and induction pathways through enzyme modulation, thus with potential to affect gene expression (11). Although it is unquestionable that the primary mechanism of action of α-tocopherol results from its antioxidative role, these other properties require further confirmation in in vivo studies (11).

It has further been highlighted that α-tocopherol might compete for absorption with other lipid micronutrients such as carotenoids, vitamins A, D and K, which, except for vitamin A, presumably occur due to common uptake pathways involving cholesterol transporters mentioned earlier (16).

## Assessment of nutrient status

Dietary intake surveys are a widely used method to assess the nutrient status of populations (12). Collected data on food consumption is converted to nutrient intake through linkage to food composition databases, which subsequently generate data on average nutrient intakes (20). Limitations of such methods are the possible underreporting of food intake and quality differences of food composition database used (12).

The determination of a daily nutrient requirement depends not only on assessing its function but also on defining a biomarker that functions as an indicator of inadequacy and captures nutrient intake changes (3). Three potential biomarkers have been discussed in the literature. In 2000, NASEM chose results from the in vitro hydrogen peroxide–induced erythrocyte hemolysis test as marker for α-tocopherol status, because increased peroxide-induced erythrocyte hemolysis correlated with increased erythrocyte fragility in α-tocopherol-deficient individuals. Based on this, the EAR for α-tocopherol in humans was set on the basis of α-tocopherol depletion and repletion studies in men using erythrocyte hemolysis as the biomarker. The current RDA of 15 mg α-tocopherol was extrapolated from that value.

Further acknowledged markers of measuring α-tocopherol status in clinical and observational studies are fasting blood α-tocopherol concentration, measured in either plasma or serum (21). It is, however, difficult to interpret plasma or serum α-tocopherol concentrations. They largely depend on plasma lipid concentrations, which are known to increase with age and consequently cause an increase in plasma carriers for α-tocopherol, in turn leading to higher circulating α-tocopherol concentrations (3). Therefore, correction for plasma lipids is warranted in subjects with high lipid levels when assessing α-tocopherol status in populations. Moreover, if both plasma lipids and α-tocopherol are abnormally low, then correction of circulating α-tocopherol concentrations for plasma lipids will yield a value indicating a normal α-tocopherol:lipid ratio (3). Other factors affecting plasma α-tocopherol concentration are gender, lifestyle, genetic variation and variation in the absorption, metabolism and excretion of α-tocopherol, obesity, metabolic syndrome or high levels of oxidative stress (11). Finally, urine or plasma α-carbocyethyl hydroxychroman (α-CEHC), which is the major metabolite of α-tocopherol following supplementation, has been suggested as an alternative biomarker of adequate α-tocopherol status. There is, however, insufficient evidence on its relationship with dietary α-tocopherol intake and saturation of body tissues with α-tocopherol (11, 22). Further, the methodology is not sensitive enough to detect low levels of α-tocopherol, and is thus not widely used (11).

## Dietary intakes in the Nordic and Baltic countries

Relevant food sources of α-tocopherol are vegetable oils, vegetable oil-based spreads, nuts, seeds and egg yolk. The α-tocopherol content is highest in sunflower oil followed by corn and rapeseed oil, olive oil and soybean oil. About half of the α-tocopherol in the diet of Finnish adults was provided by cereal, bakery products, fat spreads, oils and dressings (23). Among the EPIC study participants from the Nordic countries, added fats contributed the most to α-tocopherol intake followed by cereal, cereal products and cakes (24). Other significant sources were fruits, vegetables, fish and shellfish. In the recent dietary surveys from the Nordic and Baltic countries (20), the mean dietary intake of α-tocopherol among adult populations varied between 8.8 and 13.2 mg/day in Nordic countries and 7.8 and 13.9 mg/day in Baltic countries, with an overall average intake across countries of 12.2 mg/day. In general, all countries reached the NNR2012 levels of recommended intakes (RI) of dietary α-tocopherol, which was 10 mg/day for men and 8 mg/day for women, except Danish men and Estonian women. When comparing α-tocopherol intake across age subgroups, levels of RI were met, although slightly lower levels were reported for Danish men in general, as well as Finnish men in the age group 65–75 years. When expressed in relation to energy intake, dietary intake of α-tocopherol by adults in the Nordic countries ranged from 8.5 mg/10 MJ to 16 mg/10 MJ and in the Baltic countries from 11 mg/10 MJ to 17 mg/10 MJ. During pregnancy, intake of dietary and supplements containing α-tocopherol was usually higher (25–27). Age-specific RI levels for α-tocopherol intake in children were generally met, except in Danish and Estonian adolescents aged 14–17 years and Estonian females aged 10–13 years. No information on children or adolescents was available for Iceland and Latvia (20).

## Health outcomes relevant for Nordic and Baltic countries

### Deficiency

Vitamin E deficiency due to low dietary intake has not been described in healthy adults. However, deficiency can be caused by prolonged fat malabsorption due to genetic defects in lipoprotein transport or in the hepatic α-TTP or fat-malabsorption syndromes, such as cholestatic liver disease or cystic fibrosis (3). In adults, clinical symptoms of vitamin E deficiency, such as peripheral neuropathy, spinocerebellar ataxia and skeletal myopathy have been reported at serum α-tocopherol concentrations below 8 μmol/L (12). Vitamin E deficiency is more frequently found in children, likely due to limited stores and rapid growth (3). Specifically, premature and very low birth weight infants are at risk symptoms such as haemolytic anaemia, thrombocytosis and oedema (28).

### Toxicity

The toxicity of natural α-tocopherol is low, due to efficient metabolic control that prevents any excess accumulation of the vitamin in the body. No adverse effects have been described from intakes provided by food sources. Excess α-tocopherol can cause increased bleeding tendencies, likely as a result of interference with vitamin K (17). Further, consumption of high-dose α-tocopherol supplements (≥300 mg/d) may lead to interactions with drugs such as aspirin, warfarin, tamoxifen and cyclosporine A and may alter their activities (29).

### Chronic diseases

Studies investigating the relationship between α-tocopherol and chronic diseases rely either on dietary intake of α-tocopherol, mainly measured through a food frequency questionnaire, or self-reported supplementation or interventional supplementation with synthetic or natural α-tocopherol. A meta-analysis found that α-tocopherol supplementation was related to a reduced risk of myocardial infarction (30). Two meta-analyses focusing on vitamin E supplementation and stroke found no association (31, 32), whereas another meta-analysis concluded that those with a high dietary α-tocopherol intake had a 17% lower risk of stroke (33).

There is sparse data on α-tocopherol and its effect on diabetes. In persons with type 2 diabetes, α-tocopherol supplementation neither improved blood lipid parameters (34), nor glycaemic control (35).

Currently, there is no evidence of dietary or supplemental α-tocopherol for a protection from overall cancer (36–38), nor ovarian- (39), colorectal (38, 40) and breast cancer (41), nor prostate cancer risk (42) when considering findings from meta-analyses. Dietary, but not supplemental α-tocopherol, might be related to a lower risk of bladder (38, 43), kidney (44), as well as lung (45), uterine (46), oesophageal (47), pancreatic (48, 49) and gastric cancer (50).

There is some evidence that dietary α-tocopherol, but not supplemental α-tocopherol, might have protective effects on cognitive impairment like Alzheimer’s (51–54) and Parkinson’s disease (55). Further, dietary α-tocopherol intake reduced the risk of age-related cataract, but no association was found with supplemental α-tocopherol (56–58), neither on the risk of developing age-related macular degeneration (59). Moreover, dietary α-tocopherol was related to lower fracture risk (60). Results from a number of meta-analyses on all-cause mortality do not provide any support for recommending dietary or supplemental α-tocopherol (61–64). Although one meta-analysis reported no association with mortality with α-tocopherol supplementation ranging from doses of 23–800 IU (10–360 mg) in generally healthy individuals (62), another meta-analysis reported increased mortality risk at doses above the US RDA of 15 mg/day of α-tocopherol including trials for primary and secondary prevention (65).

## Requirement and recommended intake

There is some evidence for dietary α-tocopherol to prevent the development of certain chronic diseases, and potential beneficial effects have been observed already at low doses ranging from approximately 2–15 mg/day. However, the evidence for α-tocopherol supplementation for the prevention of cardiovascular disease and stroke is contradicting and there is no compelling evidence supporting an effect of α-tocopherol supplementation on cancer risk, other health related outcomes or overall mortality. Further, there is no evidence that α-tocopherol is protective against preeclampsia in women at either low, moderate or high risk (66, 67), nor that it prevents stillbirth, neonatal death, preterm birth, preterm or term PROM (premature rupture of membranes before labor begins) or poor fetal growth. Further research is required to elucidate the possible role of α-tocopherol in the prevention of placental abruption (66, 68). Taken together, the available scientific data suggest that there are no overall benefits of prolonged high intakes of supplemental α-tocopherol in the general population. Some literature suggests an increased risk, especially when including primary and secondary prevention studies.

Several studies from Nordic populations reported average α-tocopherol intakes ranging from 6–14 mg per day and related mean serum α-tocopherol concentrations between 23–30 μmol/L among adults (12). Only a few cases of neurological symptoms with ataxia due to α-tocopherol deficiency have been reported in the Nordic countries (69). It is not possible to directly assess adequacy of human α-tocopherol status from circulating α-tocopherol concentrations, but an inadequate status can be determined from low values (3). NASEM considered plasma levels below 12 μmol/L as deficient (7), whereas plasma levels >30 μmol/L have shown beneficial health effects (70). Although plasma levels in the Nordic countries did not exceed levels found to be beneficial, they also do not seem to fall below suggested levels of manifest or symptomatic deficiency. Thus, the available data indicate that α-tocopherol status is sufficient in the Nordic populations.

### Adults

To establish the average requirements and recommended α-tocopherol intakes among adults, the criteria used are the relationship between α-tocopherol and PUFA intake. The α-tocopherol requirement related to dietary linoleic acid, which globally is the major dietary PUFA in humans, has been calculated to be 0.4–0.6 mg of RRR-α-tocopherol per g of linoleic acid (71). The Scientific Committee on Food considered a ratio of 0.4 mg α-TE/g total PUFA to be adequate for adults (72) provided α-tocopherol does not fall below 4 mg/d for adult men and 3 mg/d for adult women.

In 2003, the Scientific Committee on Food proposed an upper level of α-tocopherol of 300 mg/d for adults, based on effects of increased intakes of α-tocopherol supplementation on the impact of blood clotting (22). In NNR 2012, the upper intake level of 300 mg/d α-tocopherol as supplement, established by the European Food Safety Authority (EFSA), was included. However, additional, long-term studies are warranted.

### Children

The RIs for infants and children are generally based on the α-tocopherol content in breast milk and the relationship between α-tocopherol and linoleic acid or total PUFA. Based on EFSA 2014, α-tocopherol content in breast milk is on average 4.6 mg/L (10.6 μmol/L) in women who do not take any supplementation (22).

Similarly to adults, the Scientific Committee on Food considered a ratio of 0.4 mg α-TE/g total PUFA to be adequate also for children (72). This ratio was shown to maintain normal plasma tocopherol levels in growing children (71).

### Pregnancy and lactation

Since there is no evidence for a beneficial effect of α-tocopherol supplementation during pregnancy and lactation, the recommendation may be based on a higher intake of energy and PUFA during these sensitive periods. The RI during lactation should also include the extra requirement to cover secretion in breast milk.

## Limitations

Some of the evidence related to chronic diseases presented in this scoping review relies on findings from observational studies, rather than randomised controlled trials. α-Tocopherol intake is commonly estimated through self-reported FFQs, which are prone to recall bias. Further, the effect of α-tocopherol cannot fully be separated from other nutritional factors.

## Funding

Funding was received from the Nordic Council of Ministers and governmental food and health authorities of Norway, Finland, Sweden, Denmark, and Iceland.
